# The Effect of Unpredictable Chronic Stress on Rare Minnow (*Gobiocypris rarus*): Growth, Behaviour and Physiology

**DOI:** 10.3390/biology11121755

**Published:** 2022-12-01

**Authors:** Chunsen Xu, Liangxia Su, Ning Qiu, Miaomiao Hou, Fandong Yu, Xinhua Zou, Jianwei Wang

**Affiliations:** 1 Institute of Hydrobiology, Chinese Academy of Sciences, Wuhan 430072, China; 2 University of Chinese Academy of Sciences, Beijing 100049, China; 3 National Aquatic Biological Resource Center, Institute of Hydrobiology, Chinese Academy of Sciences, Wuhan 430070, China

**Keywords:** rare minnow, UCS, growth performance, behaviour patterns, physiology

## Abstract

**Simple Summary:**

The laboratory fish model plays an important role in modern scientific research. Ensuring the welfare of laboratory fish is beneficial to the repeatability of experimental results. Laboratory fish will face some stressors in the process of feeding and management, but there are few studies on this part. Our study shows that unpredictable chronic stress for 7 and 14 days can lead to a significant decrease in growth and cortisol levels of laboratory rare minnow. At the same time, the behaviour pattern and neurotransmitter response changed more significantly with the increase of time. Therefore, we should try to reduce the duration and intensity of these stressors to ensure their welfare needs in daily feeding management.

**Abstract:**

Fishes often adjust their behaviour patterns and physiological responses to cope with changing environments, and different life experiences affect them differently. Fishes might adapt to short-term stress, whereas long-term unpredictable stress may lead to various adverse effects. Although some studies have constructed unpredictable stress models of fish, the effect of unpredictable chronic stress (UCS) in the laboratory is poorly understood in fishes. In the current study, we exposed adult rare minnow to an unpredictable chronic stress protocol over 7 and 14 days and measured their response in terms of growth performance, cortisol, neurotransmitter levels (DA, 5-HT, and related metabolites), and behaviour patterns to comprehensively assess the effects of UCS on laboratory rare minnow. We discovered that specific growth rates were significantly decreased, and cortisol levels were lowered in both 7-days and 14-days stress groups. In the behaviour test, the activity level of the 14-days stress group increased, but there was no significant difference in the number of crossings to the center areas, time spent in the center areas, or the speed. In addition, the levels of DA and 5-HT did not change in the stress groups, but the DOPAC and 5-HIAA levels in the 14 days stress group were significantly higher than those in the control group. These results suggested that UCS influences rare minnow growth performance, behaviour patterns, and cortisol levels, and similar stress should be minimised in the laboratory.

## 1. Introduction

Fishes often face a changing environment and various stresses, especially in the wild, such as abiotic factors (changes in the physical and chemical properties of water bodies) and biological factors (predator pressure) [1]. Although most stressors can be eliminated in the laboratory, some stresses (capture, handling, and restraint) are still unavoidable [2,3]. In the wild environment, fishes can escape or find shelter to stay away from the stressor and restore the homeostasis of the internal milieu more quickly. However, in the laboratory, the stress response of fish may last longer because of space constraints and lack of shelter [4,5].

Stress usually refers to changing one’s condition in response to a changing environment, which is a physiological process that adapts and maintains the homeostasis of the internal environment [6]. The results of stress are often related to species and the external environment [1]. Once the source of stress stops, it will often return to a steady state if the stress is short-lived, which is often adaptive [7]. However, the stress in the laboratory is often long term, chronic, and unpredictable. If fishes are in such an environment for a long time and cannot deal with it effectively, it will often lead to a wide range of effects on the body.

When fishes encounter stress, the hypothalamic-pituitary-interrenal tissue (HPI) axis and neurotransmitter activity are generally considered the primary stages of neuroendocrine regulation. Subsequent secondary reactions will affect immune function, enzyme activity, and blood parameters, finally leading to changes in behaviour, reproductive ability, growth, and survival at the organism level [8,9,10,11]. Brain serotonergic system activity, dopaminergic system activity, and cortisol levels are considered commonly used indicators of stress in fish [5,12,13,14,15].

Although some studies have simulated the effects of unpredictable stress on some experimental animals [16,17], they are more likely to be used to construct chronic stress models. In fact, some stressors are almost unlikely to appear in the laboratory (such as the emergence of predators). Relatively few attempts have been made to quantify the effects of common stressors that occur in the laboratory, which are closely related to animal welfare and the validity of experimental results.

In this study, we used possible stressors in the laboratory (air exposure, chase, crowding, low water level, and fasting) to simulate unpredictable chronic stress (UCS). First, we examined the physiological responses of chronic stressors at different times by measuring the changes in the levels of cortisol, dopamine, serotonin, and their metabolites. Second, we explored the changes in the behaviour of the whole animal exposed to unpredictable stressors at different times by assessing the behavioural differences between exposed and unexposed fishes. Finally, we used the growth performance to reflect a comprehensive effect of UCS on the rare minnow (*Gobiocypris rarus*).

Rare minnow, a small cyprinid fish, has been widely used as a native laboratory fish for chemical testing and research on disease, toxicology, behaviour, and genetics [18,19,20,21,22,23,24,25]. For a laboratory animal, serial standard drafts have been established for a rare minnow, including controls on pathogens, heredity, environment, and nutrition. However, UCS has not been involved in standard drafts. This study aimed to investigate the effect of UCS on the growth, behaviour patterns, and physiological status of laboratory rare minnows to explore whether similar stressors can be minimised in daily management.

## 2. Material and Methods

### 2.1. Fish Culture and Handling

Rare minnow (*Gobiocypris rarus*) were provided by the National Aquatic Biological Resource Center, NABRC. The whole experiment was finished in a NABRC’s laboratory. Based on the experimental design and sampling requirements, ninety fish (total length: 38.85 ± 2.47 mm, body weight: 0.52 ± 0.07 g, ~six month old) were randomly and equally placed into nine plastic tanks (length: 40.0 cm, width: 25.0 cm, and height: 20.0 cm), and ten rare minnows were raised in each tank. All individuals belonged to the same recirculating aquatic housing system equipped with multistage filtration including activated filter stone, filter sponge, and UV sterilisation. The tanks were arranged into three treatments: control and stress groups (7 or 14 days of UCS). The details of UCS are given in Table 1. Crowding, Chasing, Low water level to dorsal and food deprivation were conducted in the system feeding tank. A net was used to expose the fish to air. The control group had no handling. Test fish were reared in the tanks for 7 or 14 days until they were sampled or used in behavioural experiments.

During the rearing period, the water depth was maintained at 16 cm, and the water flow rate was maintained at 700 mL/min. The light/dark cycle was controlled as 12:12 h. HACH30D was used to determine the water parameters. Water temperature was maintained as 26.6 ± 0.3 °C. The pH was between 7.56 to 8.22. Fish were fed enough commercial dry pellet (crude protein ≥ 35%, crude fat ≥ 3.0%, crude fibre ≤ 8.0%, crude ash ≤ 15%, moisture ≤ 10%, calcium ≥ 12%, phosphorus ≥ 0.6%, and lysine ≥ 1.5%) twice daily at 10:00 am and 4:00 pm. No injured or dead individuals were found during the whole rearing period, and the rest of the individuals were transferred back to the NABRC at the end of the experiment.

### 2.2. Behavioural Studies

**Figure 1:**
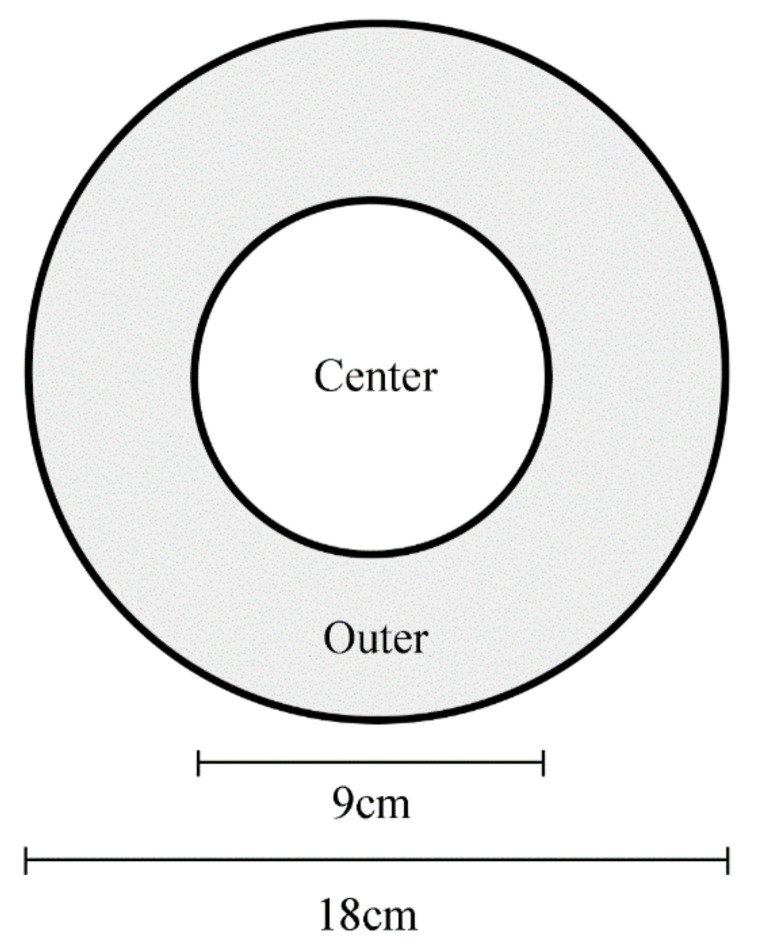
Top view of the open field test.

The open-field test was performed according to a previous study [26]. Less time spent in the center and crossing the center fewer times reflects more anxiety-like behaviour [27,28]. The round glass container was divided into the equal center and outer areas, and grey stickers were affixed around the cylinder wall to prevent external interference (Figure 1). The following parameters were counted through the Zebralab system (Zeb-view, France, For details, please see: https://www.viewpoint.fr/zh_CN/search/%E6%96%91%E9%A9%AC%E9%B1%BC (accessed on 1 November 2022). Speeds < 0.5 cm/s, 0.5–3 cm/s, and >3 cm/s were defined as inact, small movement, and large movement, respectively. Indur, smldur, and lardur represented the time spent in inact, small, and large movements, respectively. Inadist, smldist, and lardist represented the distance spent in inact, small, and large movements, respectively. The times of crossing to the center and staying in the center area were counted manually by watching the video back. Overall, 18 fish (six from each replicate tank) were randomly selected and tested one by one. The time in the central area is defined as the time taken from the entry of the whole body to the time it leaves. Test fish were gently put into the container, and fresh system water was added after each behaviour test. After 2 min of adaptation, data were collected through the camera within 8 min.

### 2.3. Sampling and Measuring of Physiological Parameters

To assess the long-term effects of stress, we conducted behaviour tests and sampling of physiology test on the 8th and 15th days of stress, respectively. In addition, to minimise the impact of the pheromone generated by the stress group (7 or 14 days of UCS) on the control group through the circulatory system, the behaviour and physiology experiment was conducted on the control group on the 8th day [29,30].

The fish used for behaviour and physiology experiments were independent. In order to meet the sampling requirements, four fish from the same tank (three replicated tanks) were sacrificed in ice water [16]. After measuring the total body length and weight, each fish was dissected on the ice immediately. The whole brain was used for determining the levels of neurotransmitters, including DA, DOPAC,5-HT,5-HIAA, and brain protein. The rest of the body was used for determining the cortisol levels. All procedures performed in this study were approved by the Institutional Animal Care and Use Committee of the Institute of Hydrobiology, Chinese academic of sciences (IHB/LL/2020025).

Four fish brains tissues or two bodies were mixed to form one sample. Mixed brain or body samples were homogenised in cold PBS (9 × weight, pH 7.4) and centrifuged in a refrigerated centrifuge of 3000 rpm (4 °C) for 20 min. The supernatant were collected in tubes and used for subsequent experiments. The cortisol, DA, DOPAC, 5-HT,5-HIAA, and brain protein concentrations were measured using fish-special commercial ELISA Assay Kit (Jiangsu Meimian Industrial Co., Ltd., Yancheng, China) according to the manufacturer’s instructions.

### 2.4. Data Analysis

The growth performance was evaluated by specific growth rate (SGR).SGR% = 100 × [ln(BW_f_) − ln(BW_i_)]/T, where BW_i_ represents body weight at the beginning of the experiment, BW_f_ represents body weight at the end of experiment, and T(d) represents the rearing days. Considering that fasting is included in the stress protocol, T(7d) is equal to 5.5, and T(14d) is equals to 11. Neurotransmitter levels were normalised to total brain protein weight (expressed as ng/g of brain protein), and cortisol level was normalised to body weight (expressed as ng/g body weight).

The Shapiro–Wilk test was used to determine conformity with the normal distribution.

If the data obeyed normal distribution, one-way ANOVA was used to analyse the differentiation between any groups. Bonferroni or Tamhane’s T2 was used for post hoc analysis when meeting the assumption of the Levene test or not, respectively. If the data did not obey normal distribution, the Kruskal–Wallis test was used. All statistical analyses were performed using SPSS 25.0.Statistical significance was defined by *p* < 0.05.

## 3. Result

### 3.1. Growth

**Figure 2:**
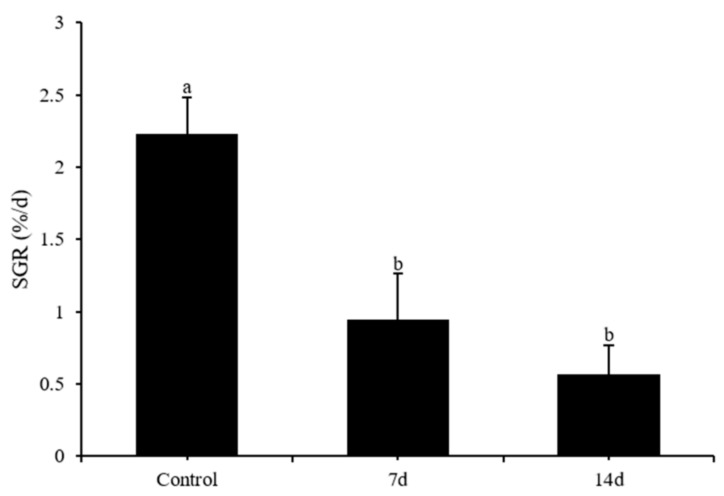
Effects of 7 and 14 days of the UCS protocol on the specific growth rate (SGR) in the rare minnow. Data are expressed as mean ± SD (n = 3). The different letters in the bar indicate significant differences between the two groups (*p* < 0.05).

After different periods of UCS, the SGR of the control group was 2.23 ± 0.25, whereas the UCS of 7 and 14 days was 0.94 ± 0.32 and 0.56 ± 0.2, respectively (Figure 2). The control group had a significantly higher SGR than the stress group (Bonferroni post hoc: *p* < 0.01 control vs stress groups), whereas no difference was observed between 7 and 14 days of UCS (Bonferroni post hoc: *p* = 0.59).

### 3.2. Behavioural Parameters

**Figure 3:**
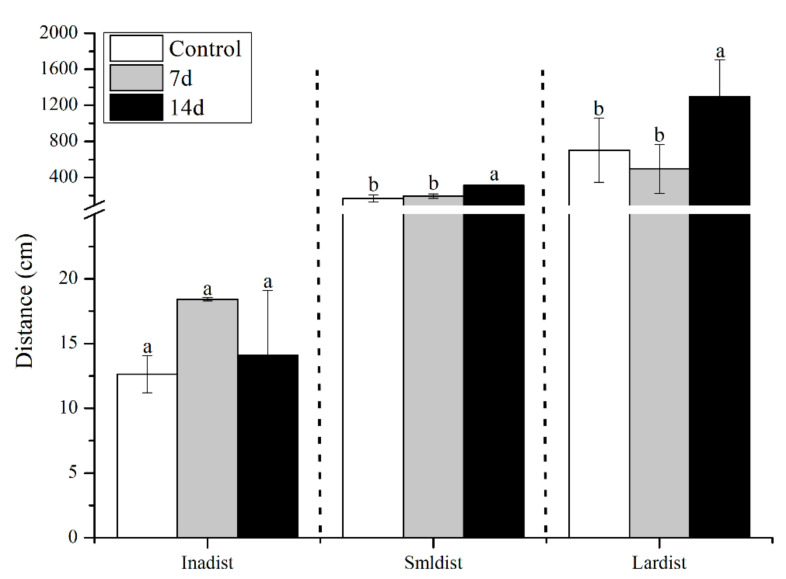
Effects of 7 and 14 days of the UCS protocol on inadist, smldist, and lardist in the rare minnow. Data are expressed as mean ± SE (n = 18). The different letters in the bar indicate significant differences between the two groups (*p* < 0.05).

**Figure 4:**
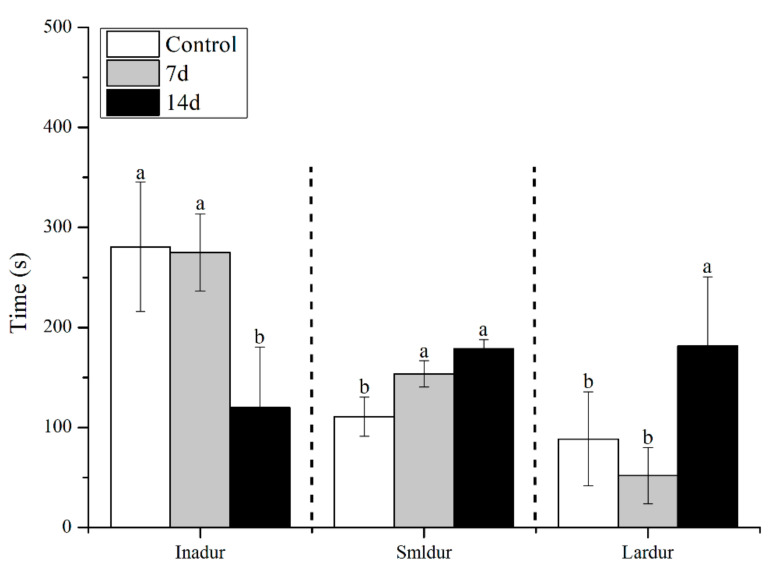
Effects of 7 and 14 days of the UCS protocol on inadur, smldur, and lardur in the rare minnow. Data are expressed as mean ± SE (n = 18). The different letters in the bar indicate significant differences between the two groups (*p* < 0.05).

**Figure 5:**
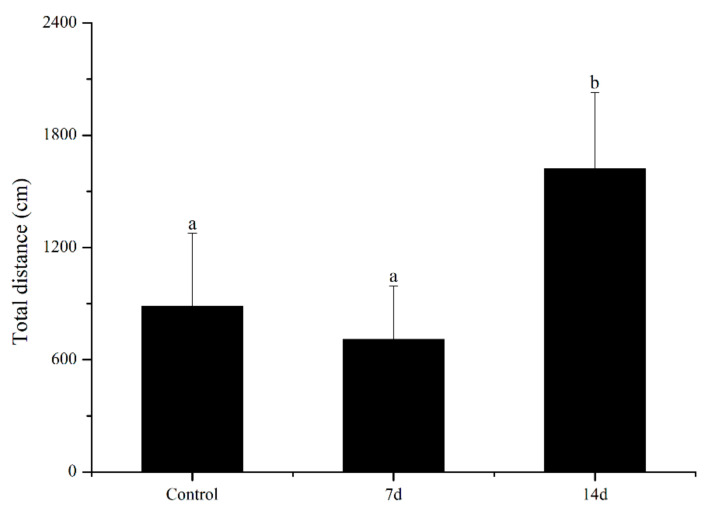
Effects of 7 and 14 days of the UCS protocol on the total distance in the rare minnow. Data are expressed as mean ± SE (n = 18). The different letters in the bar indicate significant differences between the two groups (*p* < 0.05).

**Figure 6:**
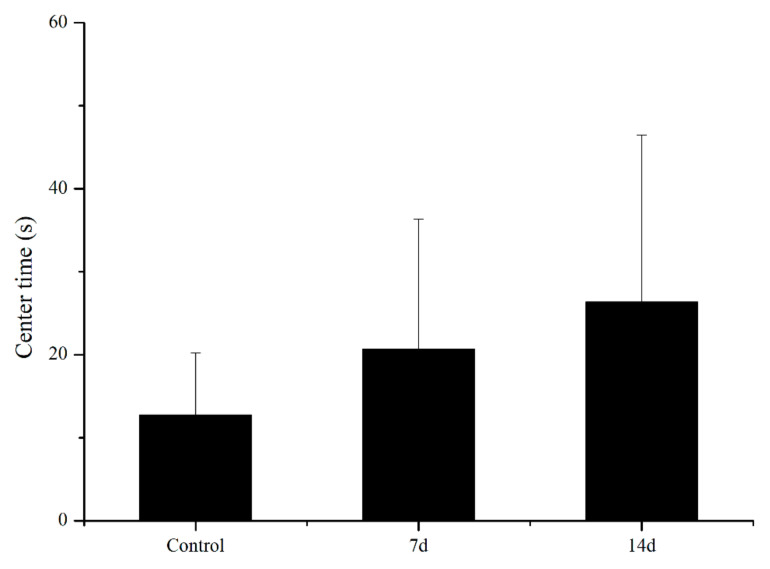
Effects of 7 and 14 days of the UCS protocol on center time in the rare minnow. Data are expressed as mean ± SE (n = 15–18).

**Figure 7:**
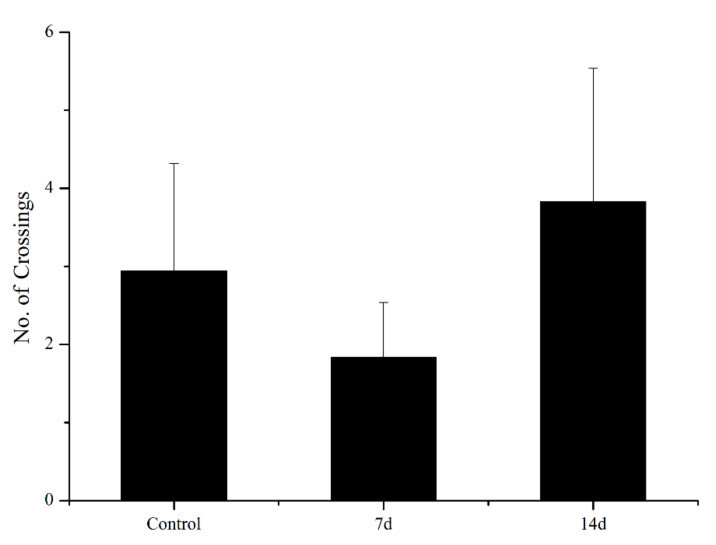
Effects of 7 and 14 days of UCS protocol on several crossing the center in the rare minnow. Data are expressed as mean ± SE (n = 15–18).

**Figure 8:**
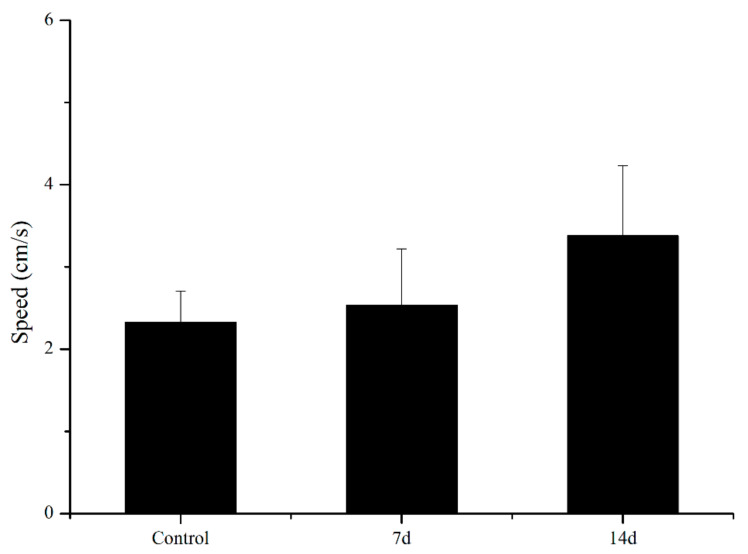
Effects of 7 and 14 days of the UCS protocol on speed in the rare minnow. Data are expressed as mean ± SE (n = 18).

UCS led to behavioural alterations in behaviour patterns of the open field task, as shown in Figure 3 and Figure 4. UCS did not change inadist between groups (Kruskal–Wallis: control vs stress *p* = 0.09), whereas the smldist (Bonferroni post hoc: 14 days of UCS vs. control and 7 days of UCS: *p* < 0.01) and lardist (Kruskal–Wallis: 14 days of UCS vs. control: *p* = 0.03; 14 days of UCS vs. 7 days of UCS: *p* = 0.03) increased significantly after 14 days of UCS. Both 7 and 14 days of UCS significantly increased smldur in the tank (Bonferroni post hoc: control vs. 14 days UCS: *p* = 0.038; 7 days UCS vs. 14 days UCS: *p* < 0.01). Inadur was lower after 14 days of stress (Bonferroni post hoc: 14 days of UCS vs control and 7 days of UCS: *p* < 0.01). Lardur increased after 14 days of stress (Kruskal–Wallis: control vs. 14 days UCS *p* = 0.03; 7 days of UCS vs. 14 days UCS: *p* = 0.01). Moreover, the total distance was significantly increased after 14 days of UCS (Figure 5) (Kruskal–Wallis: control vs. 14 days UCS: *p* = 0.01; 7 days of UCS vs. 14 days UCS: *p* = 0.02). Time spent in the central area (Kruskal–Wallis, *p* = 0.31), number of crossings to the center area (Kruskal–Wallis, *p* = 0.09), and speed (Kruskal–Wallis, *p* = 0.15) did not change between groups (Figure 6, Figure 7 and Figure 8).

### 3.3. Physiological Changes

**Figure 9:**
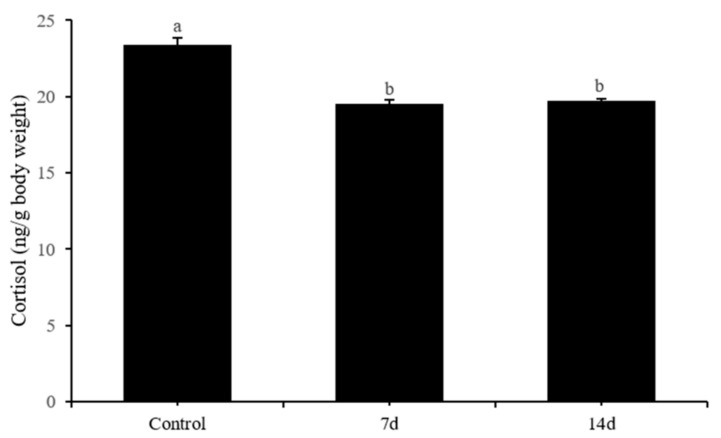
Effects of 7 and 14 days of the UCS protocol on cortisol levels in rare minnow. Data are expressed as mean ± SD (n = 3). Three pooled samples of two fish each from each treatment. The different letters in the bar indicate significant differences between the two groups (*p* < 0.05).

**Figure 10:**
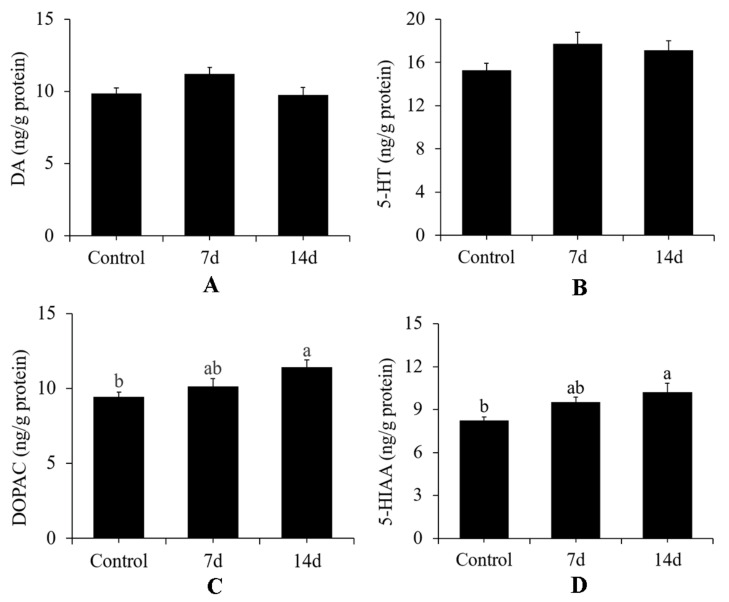
Effects of 7 and 14 days of the UCS protocol on neurotransmitter levels in rare minnow. (**A**) DA, (**B**) 5-HT, (**C**) DOPAC, and (**D**) 5-HIAA. Data are expressed as mean ± SD (n = 3). Three pooled samples of four fish each from each treatment. The different letters in the bar indicate significant differences between the two groups (*p* < 0.05).

Post hoc analysis revealed that both 7 and 14 days of UCS significantly reduced cortisol levels (Figure 9) (Bonferroni post hoc: *p* < 0.001). In contrast, DA (Bonferroni post hoc: control vs. 7 days of UCS: *p* = 0.08; control vs. 14 days of UCS: *p* = 1; 7 days of UCS vs. 14 days of UCS: *p* = 0.06) and 5-HT (Bonferroni post hoc: control vs. 7 days of UCS: *p* = 0.1; control vs. 14 days of UCS *p* = 0.24; 7 days of UCS vs. 14 days of UCS: *p* = 1) levels exhibited no differences between groups. Dopac (*p* = 0.01) and 5-Hiaa (*p* = 0.01) levels were higher after 14 days of UCS compared with the control group (Figure 10).

## 4. Discussion

### 4.1. Effect of UCS on Growth and Cortisol Levels

In the daily feeding, we observed that the rare minnow in the stress group tended to stay on the other side of the feeding tank, whereas the control group took the initiative to swim to the feeders to wait for feeding. In addition, we also found that the control group could eat all the feed each time, but the stress group had more feed left in the feeding tank, suggesting a decrease in food intake in the stress group. Our results revealed that the specific growth rate decreased significantly after UCS for 7 and 14 days compared with the control group, and the decrease in body weight was the most consistent response induced by variable stressors [31]. No significant difference was observed between 7 and 14 days of UCS, indicating that the rare minnow has a certain degree of adaptation at the growth level.

Studies on the effect of unpredictable stressors on fish cortisol level have yielded contradictory results. For example, zebrafish and Atlantic salmon had higher cortisol level after various stressors [16,17,32], whereas no significant differences were found in female three-spined sticklebacks [1]. There is no significant difference in cortisol level between UCS for 7day and 14 days, which might point to a certain degree of adaptation of the rare minnow. After 7 and 14 days of UCS, the cortisol level in rare minnows decreased significantly. The decrease in cortisol levels caused by long-term chronic stress may be related to the hypoactivity of the HPI axis caused by exaggerated negative feedback after the initial hyperactivity of the HPI axis [33]. Evidence from the literature suggests that both elevated and decreased baseline glucocorticoid concentrations can have negative health consequences [34,35]. Although widely used as indicators of a stressed state in various taxa [36], glucocorticoid hormones exhibit a complex pattern of biosynthesis and metabolic clearance and uptake. This pattern can change on a diurnal and more long-term scale [37,38]. The differences between various studies further emphasise the importance of considering multiple non-hormonal indices of stress, including changes in behavioural phenotypes, when assessing the effects of stress conditions [10].

### 4.2. Effect of UCS on Behaviour Patterns

The open-field test is one of the most frequently used behaviour tests in animal research. It has been developed to measure anxiety-like behaviour, exploration, activity/locomotion, spatial preferences, and related behaviour patterns in rodents and fish [39,40,41,42,43]. Behavioural patterns changed after UCS for 7 and 14 days. More crossings to the center and more time in the center are related to lower levels of anxiety. Our results revealed that neither the number of crossings nor the time spent in the central area changed after UCS. It is notable that due to technical reasons, the three videos of the control and 7 days of UCS were lost, which might affect the above results.

Although the speed of the UCS group tended to increase, no significant difference was observed. In the control group, the behavioural patterns of rare minnows were mainly inact and small movements. They spent more time in small movement after UCS of 7 days. The pattern of behaviour was further changed after 14 days of UCS, which was shown by the significant increase in both large movement time and large movement distance compared with the control and 7 days of UCS. The movement level increased significantly after UCS for 14 days, which is inconsistent with previous studies on zebrafish [44]. In fact, there are complicated behaviour patterns in response to stressors among various species, which might be related to the different coping style [44,45,46,47].

In contrast to the reactive style, increased locomotor activity is often classified as a proactive coping style when exposed to a novel environment, principally based on predictions [48]. After facing stress several times, rare minnow in the 14 days UCS group may regard the novel tank as a sign of more danger and increase their activity to escape this environment.

### 4.3. Effect of UCS on Neurochemical Levels

In addition to characterizing growth performance, cortisol levels, and behavioural patterns in rare minnow, we measured the levels of neurochemicals from the whole-brain, including dopamine and serotonin, in the three groups. The dopaminergic system is usually correlated with the reward and motor function of animals [49,50,51,52]. The serotonergic system is usually involved in the regulation of various physiological functions of animals, such as aggressive behaviour, anxiety, and depression, and especially plays important roles in emotion regulation [53,54,55,56]. In rodents, UCS has been shown to reduce 5-HT and DA levels in whole-brain tissue samples [57]. In contrast, we found that UCS did not change DA and 5-HT levels significantly in this study, which might be related with the shorter experiment cycles. As metabolites of dopamine and serotonin, the contents of Dopac and 5-HIAA increased with the increase of stress time, indicating that UCS accelerated the metabolism of dopamine and serotonin. Although studies have shown that DA and 5-HT levels in specific brain regions decrease after UCS, almost as many studies have shown no effect in rats or mice [58]. In the current study, we did not distinguish between different brain regions, so we could not determine the specific responses of different brain regions to UCS. We did not observe the relationship between the changes in neurotransmitters and those in behavioural patterns, which might be related to other neurotransmitter systems [59], but this needs further investigation in the future.

## 5. Conclusions

In summary, in this study, we measured the growth performance, behavioural patterns, and physiological responses of rare minnow to different periods of UCS. Our results suggested that the specific growth rate and cortisol levels significantly decreased in the stress groups. Additionally, we observed that UCS significantly changed the behavioural patterns, Dopac, and 5-Hiaa of rare minnows, which further changed with increasing stress time. A decreases in growth, as an integrated embodiment of various physiological and ecological responses, often represents the deterioration of the external environment. Although our stress protocol could not fully represent the actual situation in the laboratory, the more accurate selection of stressors can provide some reference for laboratory management. Overall, our study provides evidence that similar stressors in our study should be minimised in daily management to prevent adverse effects on laboratory fish.

## Figures and Tables

**Table 1:** Protocol of unpredictable chronic stress. Chasing: use a net to chase the fish. Air exposed: exposed the fish to air. Low water level to dorsal: lower the water level in the rearing tank to the dorsal fin. Crowding: put the 10 fish into the net (14 × 14 × 14 cm).

Weeks	Monday	Tuesday	Wednesday	Thursday	Friday	Saturday	Sunday
Week 1	Food deprivation	9:46	11:03	9:40	9:23	13:30	13:00
		Crowding (2 min)	Air exposed (20 s)	Chasing (1 min)	Low water level to dorsal (5 min)	Chasing (1 min)	Crowding (2 min)
	16:20	17:14	Food deprivation	15:09	15:37	Food deprivation	14:42
	Chasing (1 min)	Low water level to dorsal (5 min)		Crowding (2 min)	Air exposed (20 s)		Low water level to dorsal
Week 2	Food deprivation	8:40	Food deprivation	9:10	12:45	11:17	10:32
		Chasing (1 min)		Air exposed (20 s)	Low water level to dorsal (5 min)	Crowding (2 min)	Air exposed (20 s)
	18:30	17:10	17:17	17:17	20:00	Food deprivation	15:43
	Air exposed (20 s)	Low water level to dorsal (5 min)	Crowding (2 min)	Chasing (1 min)	Chasing (1 min)		Low water level to dorsal (5 min)

## Data Availability

Data and calculation tools are available from the corresponding author upon reasonable request (wangjw@ihb.ac.cn).
